# Escalated radiation and prophylactic extended field nodal irradiation are beneficial for FIGO IIIB cervical cancer patients’ prognosis

**DOI:** 10.1186/s13014-018-1172-1

**Published:** 2018-11-20

**Authors:** Qingyu Meng, Weiping Wang, Xiaoliang Liu, Xiaorong Hou, Xin Lian, Shuai Sun, Junfang Yan, Zhikai Liu, Zheng Miao, Ke Hu, Fuquan Zhang

**Affiliations:** Department of radiation oncology, Peking Union Medical College Hospital, Chinese Academy of Medical Sciences and Peking Union Medical College, Beijing, People’s Republic of China

**Keywords:** FIGO IIIB, Cervical cancer, EQD2, IMRT, Prophylactic extended field irradiation

## Abstract

**Background:**

Currently, the standard treatment for locally advanced cervical cancer patients is concurrent chemoradiotherapy. Here we aim to evaluate therapeutic efficacy, treatment failure, toxicity and prognostic factors for FIGO IIIB cervical cancer patients.

**Methods:**

A comprehensive retrospective analysis was performed to understand various factors which contribute to IIIB cervical cancer prognosis. In total 223 well defined patients were assigned according to their pathological subtype, age, pre-treatment HGB level, tumor size, pelvic lymph node (LN) metastasis, para-aortic LN metastasis as well as external irradiation technologies, treatment duration, point A EQD2 dose and concurrent chemotherapy cycles. We then performed correlation studies of these factors and OS, DFS, LCR, DMFS using univariate and multivariate analysis respectively.

**Results:**

We managed to achieve 207 (92.8%) complete response (CR) and 16 (7.2%) partial response (PR) with acceptable adverse effects. Notably, the 5 years OS, DFS, LCR, DMFS for these patients were 61.1, 55.2, 83.6 and 66.4% respectively. Importantly, our studies suggest that escalated point A EQD2 can significantly improve OS, DFS and LCR for FIGO IIIB cervical cancer patients, furthermore, patients without para-aortic LN metastasis who received prophylactic extended field irradiation have significant survival advantage for DFS and a tendency to improve OS and DMFS.

**Conclusions:**

Our results suggest that FIGO IIIB cervical cancer patients should receive higher EQD2 (≥98Gy_10_) radiotherapy, moreover, patients without para-aortic LN metastasis should receive prophylactic extended field nodal irradiation to improve prognosis.

**Electronic supplementary material:**

The online version of this article (10.1186/s13014-018-1172-1) contains supplementary material, which is available to authorized users.

## Background

Cervical cancer is the fourth most common cancer in women worldwide, with approximately 528,000 newly reported cases and 266,000 death cases every year [1, 2]. Currently, the International Federation of Gynecology and Obstetrics (FIGO, http://www.figo.org/) clinical staging criteria is the standard for cervical cancer classification, according to which local advanced cervical cancer refers to the Ib2-IVa stages. Specifically, FIGO IIIB cervical cancer patients usually exhibit parametrial invasion which has extended to the pelvic wall. Moreover, patients usually display hydronephrosis and impaired renal function [3]. Nearly 25% of local advanced cervical cancers are defined as IIIB cervical cancer [4]. As the determination of IIIB cervical cancer is usually supported by pelvic and/or abdominal aortic lymph node metastasis [5], the prognosis for IIIB cervical cancer patients is generally unfavorable; previous studies have indicated that the local control failure can be varied from 10.63 to 41% [4–7], which could be as high as 50% for patients with bilateral uterine involvement.

The classical treatment strategy for FIGO IIIB cervical cancer patients is concurrent chemoradiotherapy (CCRT) [5, 6]. Notably, the intensity modulated radiotherapy (IMRT) is now the most widely used clinical radiotherapy technology which can deliver high dose internal irradiation while causing significantly less radiation-related tissue damage, with its unique dosimetric distribution feature, IMRT can reduce the radiation to organs at risks (OARs) without affecting the dose distributed to clinical target volume (CTV) [7]. A combination of radiotherapy with cisplatin-based chemotherapy is recommended by the National Cancer Institute (NCI), which has been used as the standard treatment strategy for cervical cancer for almost two decades. Several studies have been performed to evaluate the clinical efficacy and to define the prognostic factors for local advanced cervical cancer [8–11]. However, such studies focusing on IIIB cervical cancer patients in Chinese populations are still lacking. Here, we retrospectively analyzed therapeutic efficacy, treatment failure, toxicity and prognostic factors for 223 FIGO IIIB cervical cancer patients who were hospitalized in the Peking Union Medical College Hospital (PUMCH) from 2000 to 2014.

## Methods

### Patient characteristics

In total 223 FIGO IIIB cervical cancer patients were retrospectively analyzed in this study. Patients’ age was from 29 to 79 years old, with a median age of 50 years old. The clinical stage was determined by clinical checkup combined with biopsy analysis before the first treatment and supported by CT or PET imaging in some cases. We then divided these patients not only based on their clinical characteristics, but also according to the treatment they received. All detailed information is summarized in Table 1.

**Table 1:** General patients’ information

Character	Group definition	Case	Ratio (%)
Age	≥65	24	10.7
	< 65	199	89.3
Pathology type	Squamous	206	92.3
	Adenocarcinoma, Adeno/squamous Carcinoma	17	7.7
Tumor size	≤4 cm	48	21.5
	> 4 cm	175	78.5
HGB prior treatment	< 110 g/L	69	30.9
	≥110 g/L	149	66.8
	N.A.	5	2.2
Pelvic LN metastasis	w/t	82	25.9
	w/o	141	74.1
Para-aortic LN metastasis	w/t	31	13.9
	w/o	192	86.1
Concurrent chemotherapy	≥4 cycles	155	69.5
	< 4 cycles	45	20.10
	N.A	21	9.4
Radiotherapy	3D-CRT	48	21.5
	IMRT	175	78.5
EQD2 (point A)	22-90Gy_10_	30	13.4
	90–98 Gy_10_	54	24.2
	≥98Gy_10_	139	62.3
Therapy duration	≤63 days	175	78.5
	> 63 days	48	21.5
Prophylactic extended field irradiation	w/t	107	48.0
	w/o	85	38.1

### Radiation therapy

All patients received both external beam radiation therapy (EBRT) and intracavitary brachytherapy (ICBT) when the patient’s health condition was allowed.

The radiation treatment was carried out as previously described [12]. The EBRT technologies included three-dimensional conformal radiotherapy (3D-CRT) and intensity modulated radiation therapy (IMRT). Forty-eight patients received 3D-CRT. Briefly, by using 15MV-X rays, we applied box irradiation technology with a total dose of 36-40Gy_10_ fractioned in 20 times followed by 5 fractions of a total 10Gy_10_ irradiation. The patients’ bladders and rectums were protected by a 4 cm central lead block. The other 175 patients received IMRT with a total dose of 45–50.4Gy_10_ fractioned for 25 to 28 times using 6MV-X rays which could cover 95% PTV (1.8Gy_10_/day, 5 days a week, 5 to 6 weeks). For patients with lymph nodes metastasis, the dosage was increased to 56-60Gy_10_ and an additional dose for proximal uterus area was administrated with a total 10Gy_10_ divided in 5 fractions.

The ICBT usually started 3 weeks after EBRT and was given once or twice a week. The standard protocol for ICBT was a cumulative dose of 36Gy_10_ prescribed to point A in 5 to 7 fractions according to the International Commission on Radiation Units and Measurements (ICRU) report 38. CT imaging was performed to support real-time treatment plans determination after the applicator implantation. The irradiation dose for rectum and bladder was strictly controlled as less than 70% of the point A. For the current study, the range for prescribed point A dose was from 22.5 to 130.4 Gy_10_ (EBRT from 10 to 70.2Gy_10_ and ICBT from 6 to 60Gy_10_).

### Concurrent chemotherapy

One hundred fifty-five patients received more than 4 cycles of concurrent chemotherapy, 68 patients were treated with less than 4 cycles including 21 patients who didn’t receive any chemotherapy due to personal reasons. Patients diagnosed as squamous were treated with a weekly cisplatin-based regimen at a dose of 40 mg/m^2^/week for 4 to 6 weeks; for those adenocarcinoma patients, we applied PF regimen in addition which included cisplatin 70 mg/m^2^ on day 1 and fluorouracil 1000 mg/m^2^ from day 1 to day 4. The PF regimen was given every 3 weeks for a total of 1–2 cycles. When the treatment was finished, the outcome was evaluated according to guidelines proposed previously [13].

### Toxicity and adverse effect assessment

All the patients were monitored for toxicities and adverse effects every week during the treatment. The severity of acute complications is classified according to the Common Terminology Criteria for Adverse Events (CTCAE v2.0) (https://ctep.cancer.gov/protocolDevelopment/electronic_applications/docs/ctcv20_4-30-992.pdf). Late complications were graded according to the RTOG/EORTC 1987 toxicity scales [14].

### Follow-up

All patients were required to have a review check every 3 months during the first 2 years after the final treatment, and twice a year during the third to fifth year after the treatment, and once a year starting from the 5th year after the last treatment. The review check includes blood biochemistry, SCC Ag, gynecological examination, pelvic MRI, chest and abdomen enhanced CT. The last follow up for the current study was carried out in May 2018.

### Statistics analysis

Overall survival (OS) is defined as the time from the start of treatment to the date of death or to the date of censoring. Disease-free survival (DFS) is defined as the time interval between the start of treatment and the detection of recurrence, metastasis or death. Local control rate (LCR) is defined as the percentage of the arrest of cancer growth at the site of origin. Distant metastasis-free survival (DMFS) is defined as the beginning of radiotherapy to the detection of distant metastasis or distant metastasis -related death. OS, DFS, LCR and DMFS were calculated with the Kaplan-Meier method by using SPSS 17.0 statistical software and compared using the log-rank test. Log-rank method was also used to perform univariate analysis, when the factor was found significant (*P* < 0.05), the Cox regression model was used to execute multivariate analysis. *P* value < 0.05 was considered statistically significant.

## Results

### Treatment outcome evaluation

**Fig. 1:**
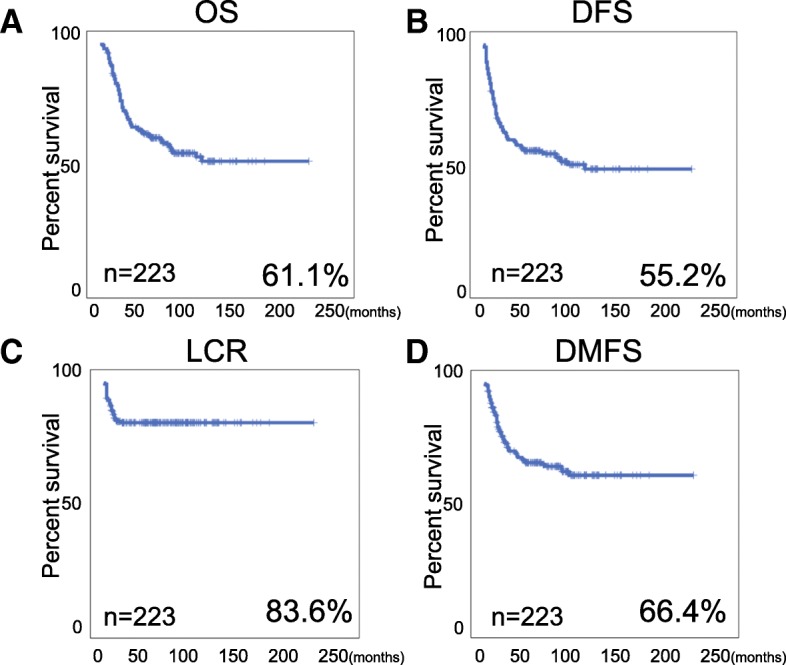
An overview of 5 years survival of FIGO IIIB cervical cancer patients treated with CCRT. The Kaplan-Meier survival curves for overall survival (OS) (**a**); disease progression-free survival (DFS); (**b**) local control rate (LCR); (**c**) and distant metastasis-free survival (DMFS); (**d**). The detail survival information is indicated separately in each figure, *n* = 223 patients for all survival analysis

For the 223 FIGO IIIB cervical cancer patients included in this study, 207 of them showed complete response (CR) after the therapy (92.8%), and only 16 patients (7.2%) exhibited partial response (PR). Importantly, the 5-year OS, DFS, LCR and DMFS were 61.1, 55.2, 83.6 and 66.4% (Fig. 1a-d), suggesting the standard concurrent chemoradiotherapy was indeed very effective for FIGO IIIB cervical cancer patients.

### Treatment failure patterns and toxicity

We observed 36 cases (16.1%) of local recurrence and 73 (32.7%) patients with distant metastasis, the details of which are summarized in Additional file 1: Table S1. Of note, there were 7 patients with both local recurrence and distant metastasis.

One major concern for CCRT treatment is the therapy-related toxicity. We have carefully evaluated both the acute toxicity and delayed toxicity. The most frequent grade 3 or 4 acute toxicity was hematological toxicity, followed by other symptoms such as frequent urination and diarrhea (Table 2). For the delayed toxicity, only few cases of complications in the urinary system and lower digestive tract were observed (Table 2). All these results indicated that CCRT was a safe therapeutic option for FIGO IIIB cervical cancer patients.

**Table 2:** Acute and delayed toxicity after treatment

	Grade 3	Grade 4
Acute toxicity (CTCEA 2.0)		
Hemoglobin	37 (16.6%)	16 (7.2%)
Leukocyte	86 (38.5%)	12 (5.3%)
Neutrophils	44 (19.7%)	11 (4.9%)
Blood platelet	26 (11.7%)	0 (0)
Frequent urination	9 (4.0%)	0 (0)
Diarrhea	16 (7.2%)	0 (0)
Delayed toxicity (RTOG/EORTC1987)		
Urinary system	7 (3.1%)	4 (1.8%)
Lower digestive tract	10 (4.5%)	5 (2.2%)

### Prognostic factors analysis

To comprehensively understand FIGO IIIB cervical cancer prognosis factors, the 223 patients were divided not only based on their clinical appearance but also according to the difference in their treatment (Additional file 1: Table S2). We then performed univariate analysis to determine prognostic factors for OS, DFS, LCR and DMFS respectively. Our data indicated that pre-treatment HGB level, tumor size, pelvic LNM, para-aortic LNM, EQD2 and concurrent chemotherapy significantly correlated with OS (Additional file 1: Figure S1 A-G); pre-treatment HGB level, tumor size, pelvic LNM, para-aortic LNM, treatment duration, EQD2 and concurrent chemotherapy cycles were significantly correlated with DFS (Additional file 1: Figure S2 A-G); moreover, tumor size, para-aortic LNM and EQD2 were highly associated with LCR (Additional file 1: Figure S3 A-C); finally, pelvic LNM, para-aortic LNM, treatment duration and concurrent chemotherapy cycles were prognostic factors for DMFS (Additional file 1: Figure S4 A-D).

Prognostic factors found to be significant (*P* < 0.05) by univariate analysis were then further analyzed using multivariate analysis. Our results suggested that pelvic LNM, para-aortic LNM, EQD2 and concurrent chemotherapy were independent prognostic factors for OS; pre-treatment HGB level, pelvic LNM, para-aortic LNM, EQD2 and concurrent chemotherapy cycles were independent prognostic factors for DFS; the independent prognostic factors for LCR included tumor size, para-aortic LNM and EQD2; and concurrent chemotherapy cycles was the only independent prognostic factor for DMFS (Table 3).

**Table 3:** Multivariate analysis for prognostic factors

Subject	HR	CI 95%	*P* value	Reference
OS				
Pelvic LN metastasis	0.558	0.363–0.858	0.008	No pelvic LN metastasis
para-aortic LN metastasis	0.381	0.232–0.624	0.000	No para-aortic LN metastasis
EQD2(point A)	3.168	1.915–5.241	0.000	EQD2 < 90Gy_10_
Concurrent chemotherapy	1.867	1.219–2.861	0.004	< 4 cycles
DFS				
Pelvic LN metastasis	0.530	0.352–0.800	0.002	No pelvic LN metastasis
Para- aortic LN metastasis	0.446	0.275–0.722	0.001	No para-aortic LN metastasis
EQD2(point A)	3.416	2.061–5.664	0.000	EQD2 < 90Gy_10_
HGB	0.652	0.435–0.979	0.039	*HGB prior treatment < 110 g/L*
Concurrent chemotherapy	1.907	1.266–2.873	0.002	< 4 cycles
LCR				
Tumor size	0.254	0.060–1.069	0.062	Tumor≤4 cm
Para- aortic LN metastasis	0.354	0.172–0.727	0.005	No para-aortic LN metastasis
EQD2(point A)	5.925	3.019–11.630	0.000	EQD2 < 90Gy_10_
DMFS				
Concurrent chemotherapy	1.874	1.160–3.028	0.010	< 4 cycles

### Higher EQD2(point A) is correlated with better treatment outcome

**Fig. 2:**
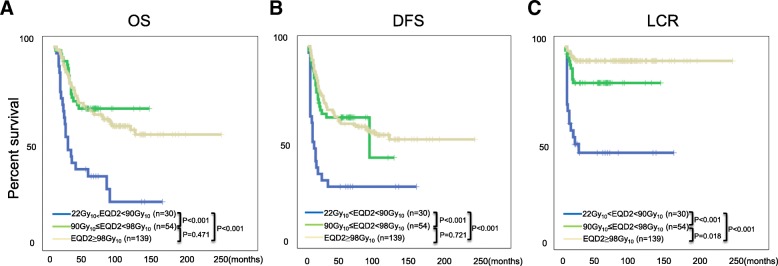
Higher EQD2 (point A) is correlated with better treatment outcome. All patients were further divided into 3 groups based on the EQD2 (point A), namely low EQD2(22Gy_10_<EQD2<90Gy_10_, blue line), medium EQD2 (90Gy_10_≤EQD2<98Gy_10_, green line) and high EQD2(≥98Gy_10_, grey line). The correlation between EQD2 (point A) and OS (**a**), DFS (**b**) and LCR (**c**) are presented. *P* values are indicated separately in the figures. *n* = 223 patients in total

As our data constantly suggested that EQD2 dosage was a detrimental factor for FIGO IIIB cervical cancer prognosis in various analysis, we further analyzed the correlation between EQD2 level and OS, DFS and LCR by distributing all the patients into high ((≥98Gy_10_), medium (90Gy_10_ ≤ EQD2 < 98Gy_10_) and low EQD2(22Gy_10_ < EQD2 < 90Gy_10_). As expected, high and medium EQD2 significantly improved patients’ OS and DFS compared to low EQD2 (Fig. 2a, b); surprisingly, we observed not only a markedly LCR improvement in patients treated with medium EQD2 compared to low EQD2, but also a significant advantage for LCR in high EQD2 treated patients compared to medium EQD2 treated patients (Fig. 2c). These data further strengthen our finding that higher EQD2 is associated with better prognosis.

### Prophylactic extended field irradiation is an important treatment option for patients without para-aortic LN metastasis

**Fig. 3:**
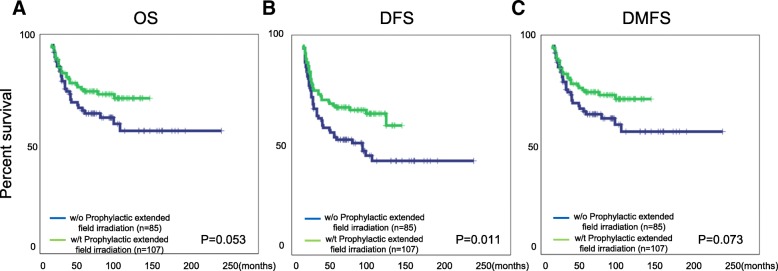
Prophylactic extended field irradiation can improve prognosis in FIGO IIIB cervical cancer patients without para-aortic LN metastasis. The patients without para-aortic LN metastasis (*n* = 192) were divided into 2 groups according to whether they were treated with or without prophylactic extended field irradiation. The survival difference in OS (**a**), DFS (**b**) and DMFS (**c**) are presented and P values are indicated in each figure

Extended field irradiation is widely used for cervical cancer patients with para-aortic LN metastasis in clinical. In our department, for those patients (192 case) who exhibited no para-aortic LN metastasis suggested by CT or PET imaging, but with other aggressive tumor features such as common iliac LN metastasis [15], tumor size> 4 cm, pelvic wall involvement in both sides, we also applied prophylactic extended field irradiation as a supplementary treatment (107 cases). Surprisingly, when compared to those non-para-aortic LN metastases patients who didn’t receive such therapies (85 cases), we found a significant survival advantage in DFS and improved OS and DMFS (Fig. 3a-c) in patients with prophylactic extended field irradiation treatment, indicating that prophylactic extended field nodal irradiation is indeed an important supplement to improve prognosis of none para-aortic LN metastasis FIGO IIIB cervical cancer patients.

## Discussion

Most cervical cancer cases can be prevented by routine screening, treatment of precancerous lesions or HPV vaccination, which dramatically decreases cervical cancer incidence and its related mortality [16]. Nevertheless, in many countries and regions which are lacking such procedures, cervical cancer is still a big threat to women as the survival ratio for advanced cervical cancer has not been significantly improved. Sangkittipaiboon et al analyzed 19 cases of stage III cervical cancer patients and revealed that both 5 years OS was 42.1% [17]. Gadducci et al reported a 5-year overall survival of 60% in 61 stage III cervical cancer patients [18], which was similar to our findings specifically in Chinese FIGO IIIB cervical cancer patients.

The treatment failure for cervical cancer therapy is usually defined as distant metastasis or local recurrence. Hong et al analyzed 1292 FIGO I-IV cervical cancer patients and observed that among 410 treatment failure cases, 82% of were reported within 2 years after therapy [19]. Of these patients, 213(52%) had distant metastasis, 162(40%) had local recurrence, and 35(8%) had both distant metastasis and local recurrence [19]. Waggoner reported that 90% of cervical cancer recurrence occurred within 3 years after the first treatment [2]. Katanyoo described that pelvic recurrence was one major treatment failure type for FIGO IIIB cervical cancer patients [20]. In our study, 36(16.1%) patients displayed local recurrence, which occurred between 6.5–25.9 months (median time was 13.3 months) after first therapy; while 73 (32.7%) patients had distant metastasis, which occurred within 30 months (median time was 12.2 months). In line with previous studies, lung was the most common metastatic target for FIGO IIIB cervical cancers. Of note, most patients who experienced treatment failure had tumors’ diameter > 4 cm, therefore, special or additional treatment are required for these patients.

The major concern for FIGO IIIB cervical cancer patient’s treatment is the side effects. Many studies have already shown that IMRT can significantly reduce cervical cancer radiotherapy-related adverse effects compared to 3D-CRT. Chen et al reported that IMRT could induce 36 and 30% digestive and urinary tract acute adverse effects respectively compared to 80 and 60% of such effects induced by 3D-CRT [7]. Similarly, the incidence of IMRT-related digestive and urinary system chronic adverse effects were 6 and 9% compared to 34 and 23% which induced by 3D-CRT [21]. Importantly, we didn’t observe any survival difference between IMRT and 3D-CRT treated patients, indicating that IMRT was a safer radiotherapy technology compared to 3D-CRT without compromising any therapeutic efficacy. Importantly, we did notice extended-field irradiation or higher dose escalation were associated with more severe side effects, but the difference were not significant when compared to those patients with only pelvic irradiation or lower dose irradiation treatment.

The radiotherapy for FIGO IIIB cervical cancer patients includes both external beam radiation therapy (EBRT) and intracavitary brachytherapy (ICBT). ICBT, which uses point A as a reference [9] to modulate the equivalent total dose in 2-Gy fractions (EDQ2) in the targeting area, is an essential treatment to cervical cancer radiotherapy. Eifel et al reported that when point A EQD2 was less than 85Gy_10_, the five-year pelvic recurrence ratio was 33%, in contrast, this ratio was only 16% when point A EQD2 was greater than 85Gy_10_ [6]. Consistently, Schmid et al performed three-dimensional intracavitary radiation therapy and found that when EQD2 for the high-risk CTV (HRCTV) was greater than 87Gy_10_, the cervical cancer local control ratio was more than 95% [11]. In this study, most patients were treated with IMRT as external radiation therapy, in addition, intracavitary radiation therapy was designed and performed according to real-time CT or PET imaging to reduce radiation-related organ damage. When patients showed no obvious contraindications, 6 fractions of 6Gy_10_ intracavitary radiation was administrated. Thus, the point A EQD_2_ for intracavitary radiation was 48Gy_10_, and the total EQD_2_ for both IMRT and intracavitary radiation for point A could reach as high as 98 Gy_10_. This dose is higher than any other previous reports [22] and we believe this why we could achieve better prognosis. Indeed, the further investigation revealed escalated EQD2(point A) was associated with better survival. However, this strategy also introduced much more acute toxicity compared to a recent reported clinical trial result in India [23], we therefore tried to overcome these complications as previously described [24, 25], for example, we prescribed leucogen or rhG-CSF to control hematological toxicity; live Combined Bifidobacterium, Lactobacillus and Enterococcus Capsules were used to rescue intestinal flora as well as sulfasalazine or mesalazine to alleviate intestinal inflammation; when necessary, we applied levofloxacin to control kidney infection. With these measures, the delayed toxicity percentage in our patients was comparable to the Indian study [23].

Extended field nodal irradiation has been widely used to treat patients with para-aortic LN metastasis and has shown great benefit for such patients [26, 27]. In our study, for those patients without para-aortic LN metastasis but showing aggressive tumor features, we also prescribed them with prophylactic extended field nodal irradiation. Our data suggested that these patients showed marked survival advantage in DFS and improved OS as well as DMFS when compared to none para-aortic LN metastasis patients who didn’t receive such treatment. Although the advantage in OS and DMFS was not significant, which might be explained as the limited sample size, the other reason was the patients who received prophylactic extended field irradiation usually showed more aggressive carcinoma features before the treatment. Therefore, albeit further investigation with a larger patient’s sample is required, we propose that prophylactic extended field nodal irradiation is beneficial for cervical cancer patients with or without para-aortic LN metastasis.

We admitted that our study also had some limitations, first, this is a single-center retrospective analysis, a multi-centers study included many more patients will be more informative; secondly, the intracavitary brachytherapy used in our study was 2 dimensional, thus we could only evaluate the dosage using EQD2, which might not be so accurate when compared to 3 dimensional intracavitary brachytherapy; thirdly, only 30 patients received less than 90Gy_10_ irradiation, this limited numbers might impair the accuracy when interpreting the correlations between higher dosage irradiation and prognosis.

## Conclusion

Our retrospective study confirms that concurrent chemoradiotherapy (CCRT) is an efficient and safe treatment for FIGO IIIB cervical cancer patients. We provide systematic understanding of various prognostic factors which contribute to IIIB cervical cancer patients’ prognosis in China. Moreover, we propose that FIGO IIIB cervical cancer patients should be treated with higher EQD2 (≥98Gy_10_) radiotherapy plus at least 4 rounds of chemotherapy when possible, and patients without para-aortic LN metastasis should be also treated with prophylactic extended field irradiation to improve prognosis.

## Additional file

Additional file 1: **Figure S1.** Univariate analysis of different prognostic factors for overall survival (OS). (A) Pathology type and OS. *P* = 0.062; (B) pre-treatment HGB level and OS. *P* = 0.018; (C) Tumor size and. *P* = 0.019; (D Pelvic LNM and. *P* = 0.001; (E) Para-aortic LNM and OS. *P* < 0.001; (F) EQD2 (Point A) and OS. *P* < 0.001; (G) Concurrent chemotherapy cycles and OS. *P* = 0.004. **Figure S2.** Univariate analysis of different prognostic factors for disease free survival (DFS). (A) pre-treatment HGB level and DFS. *P* = 0.022; (B) Tumor size and DFS. *P* = 0.044; (C) Pelvic LNM and DFS. *P* < 0.001; (D) Para-aortic LNM and DFS. *P* < 0.001; (E) Treatment duration and DFS. *P* = 0.04; (F) EQD2 (Point A) and DFS. *P* < 0.001; (G) Concurrent chemotherapy cycles and DFS. *P* = 0.005. **Figure S3.** Univariate analysis of different prognostic factors for local control rate (LCR). (A) Tumor size and LCR. *P* = 0.039; (B) Para-aortic LNM and LCR. *P* < 0.001; (C) EQD2 (Point A) and LCR. *P* < 0.001; **Figure S4.** Univariate analysis of different prognostic factors for distant metastasis free survival (DMFS). (A) Pelvic LNM and DMFS. *P* < 0.001; (B) Para-aortic LNM and DMFS. *P* < 0.001; (C) Treatment duration and DMFS. *P* = 0.018; (D) Concurrent chemotherapy cycles and DMFS. *P* = 0.024. **Table S1.** Details for treatment failure patterns. **Table S2.** Univariate analysis for prognostic factors. (DOCX 474 kb)

## Abbreviations

3D-CRT: Three-dimensional conformal radiotherapy
CCRT: Concurrent chemoradiotherapy
CR: Complete response
CT: Computed tomography
CTV: Clinical target volume
DFS: Disease-free survival
DMFS: Distant metastasis-free survival
EBRT: External beam radiation therapy
EQD2: Equivalent dose in 2 Gy fractions
FIGO: Federation of Gynecology and Obstetrics
HGB: Hemoglobin
ICBT: Intracavitary brachytherapy
ICRU: International Commission on Radiation Units
IMRT: Intensity modulated radiotherapy
LCR: Local control rate
LN: Lymph node
NCI: National Cancer Institute
OARs: Organs at risks
OS: Overall Survival
PET: Positron emission tomography
PR: Partial response
