# Supplementary material for: Escalated radiation and prophylactic extended field nodal irradiation are beneficial for FIGO IIIB cervical cancer patients’ prognosis
Source: Radiat Oncol. 2018 Nov 20;13:223. doi: 10.1186/s13014-018-1172-1 (PMC6245932; doi:10.1186/s13014-018-1172-1)
Supplement: Supplementary file 1 — Figure S1. Univariate analysis of different prognostic factors for overall survival (OS). (A) Pathology type and OS. P = 0.062; (B) pre-treatment HGB level and OS. P = 0.018; (C) Tumor size and. P = 0.019; (D Pelvic LNM and. P = 0.001; (E) Para-aortic LNM and OS. P < 0.001; (F) EQD2 (Point A) and OS. P < 0.001; (G) Concurrent chemotherapy cycles and OS. P = 0.004. Figure S2. Univariate analysis of different prognostic factors for disease free survival (DFS). (A) pre-treatment HGB level and DFS. P = 0.022; (B) Tumor size and DFS. P = 0.044; (C) Pelvic LNM and DFS. P < 0.001; (D) Para-aortic LNM and DFS. P < 0.001; (E) Treatment duration and DFS. P = 0.04; (F) EQD2 (Point A) and DFS. P < 0.001; (G) Concurrent chemotherapy cycles and DFS. P = 0.005. Figure S3. Univariate analysis of different prognostic factors for local control rate (LCR). (A) Tumor size and LCR. P = 0.039; (B) Para-aortic LNM and LCR. P < 0.001; (C) EQD2 (Point A) and LCR. P < 0.001; Figure S4. Univariate analysis of different prognostic factors for distant metastasis free survival (DMFS). (A) Pelvic LNM and DMFS. P < 0.001; (B) Para-aortic LNM and DMFS. P < 0.001; (C) Treatment duration and DMFS. P = 0.018; (D) Concurrent chemotherapy cycles and DMFS. P = 0.024. Table S1. Details for treatment failure patterns. Table S2. Univariate analysis for prognostic factors. (DOCX 474 kb) [file 13014_2018_1172_MOESM1_ESM.docx]

Table S1: Details for treatment failure patterns.

|  | *Number* | *Ratio (%)* |
| --- | --- | --- |
| ***Locoregional recurrence*** | 36 |  |
| *Local progression* | 16 | 44.4 |
| *Cervical recurrence* | 8 | 22.2 |
| *Parametrial recurrence* | 4 | 11.1 |
| *Vaginal recurrence* | 5 | 13.9 |
| *Uterus recurrence* | 1 | 2.8 |
| *Vulva metastasis* | 1 | 2.8 |
| *Pelvic recurrence* | 1 | 2.8 |
| *Inguinal lymph node metastasis* | 1 | 2.8 |
| ***Distant metastasis*** | 73 |  |
| *Lung* | 33 | 45.2 |
| *Retroperitoneal lymph node* | 13 | 17.8 |
| *Liver* | 7 | 9.6 |
| *Bone* | 9 | 12.3 |
| *Adrenal* | 2 | 2.7 |
| *Supraclavicular lymph node* | 7 | 9.6 |
| *Axillary lymph node* | 3 | 4.1 |
| *Mediastinal lymph node* | 4 | 5.5 |
| *Omentum* | 1 | 1.4 |
| *Intestinal* | 1 | 1.4 |
| *Brain* | 2 | 2.7 |
| ***Local recurrence & distant metastasis*** | 7 | N. A |

Note: some patients showed multiple subtypes of local recurrence and metastasis in different organs.

Table S2: Univariate analysis for prognostic factors.

| *Subject Classification (n)* | | *5 years’ survival (%)* | | | |
| --- | --- | --- | --- | --- | --- |
|  |  | OS | DFS | LCR | DMFS |
| *Age* | ≥65(24) | 58.3% | 50% | 70.8% | 77.8% |
|  | <65(199) | 61.4% | 55.8% | 85.2% | 65.2% |
|  |  | p=0.076 | p=0.043 | p=0.053 | p=0.586 |
| *Histological type* | Squamous (206) | 62.8% | 56.8% | 83.8% | 67.9% |
|  | Adenocarcinoma, Adeno/squamous  Carcinoma (17) | 41.2% | 35.3% | 81.6% | 49.4% |
|  |  | p=0.063 | p=0.115 | p=0.842 | p=0.192 |
| *Tumor size* | ＜4cm(48) | 79.2% | 72.9% | 93.7% | 79.0% |
|  | ≥4cm (175) | 56.0% | 50.3% | 80.8% | 62.6% |
|  |  | p=0.020 | p=0.051 | p=0.039 | p=0.131 |
| *HGB level* | ＜110 g/L(69) | 46.2% | 42.0% | 77.9% | 54.7% |
|  | ≥110 g/L (149) | 67.9% | 61.9% | 87.1% | 71.0% |
|  |  | p=0.017 | p=0.020 | p=0.079 | p=0.073 |
| *Pelvic LN metastasis* | w/t (82) | 43.5% | 36.6% | 80.0% | 45.0% |
|  | w/o (141) | 71.2% | 66.2% | 85.7% | 78.6% |
|  |  | p=0.000 | p=0.000 | p=0.296 | p=0.000 |
| *Para-aortic LN metastasis* | w/t (31) | 22.6% | 19.4% | 60.9% | 36.7% |
|  | w/o (192) | 67.4% | 61.0% | 87.3% | 70.5% |
|  |  | p=0.000 | p=0.000 | p=0.000 | p=0.000 |
| *Concurrent chemotherapy* | ≥4 cycles (155) | 65.1% | 59.8% | 85.7% | 70.7% |
|  | <4 cycles (47) | 45.8% | 35.4% | 72.2% | 54.2% |
|  |  | p=0.001 | p=0.000 | p=0.015 | p=0.005 |
| *Radiotherapy* | Conformal (48) | 68.3% | 56.3% | 84.9% | 68.6% |
|  | IMRT (175) | 59.0% | 54.8% | 83.2% | 65.8% |
|  |  | p=0.663 | p=0.942 | p=0.779 | p=0.788 |
| *EQD2*  *(point A）* | 22-90Gy_10_ (30) | 33.0% | 26.7% | 44.9% | 60.8% |
|  | 90-98 Gy_10_ (54) | 68.0% | 62.8% | 83.0% | 75.3% |
|  | ≥98Gy_10_ (139) | 64.9% | 58.7% | 92.0% | 65.0% |
|  |  | p=0.000 | p=0.000 | p=0.000 | p=0.369 |
| *Therapy*  *duration* | ≤63 days (175) | 64.7% | 59.1% | 83.2% | 70.2% |
|  | >63 days (48) | 48.2% | 40.8% | 85.0% | 52.0% |
|  |  | p=0.117 | p=0.044 | p=0.785 | p=0.020 |


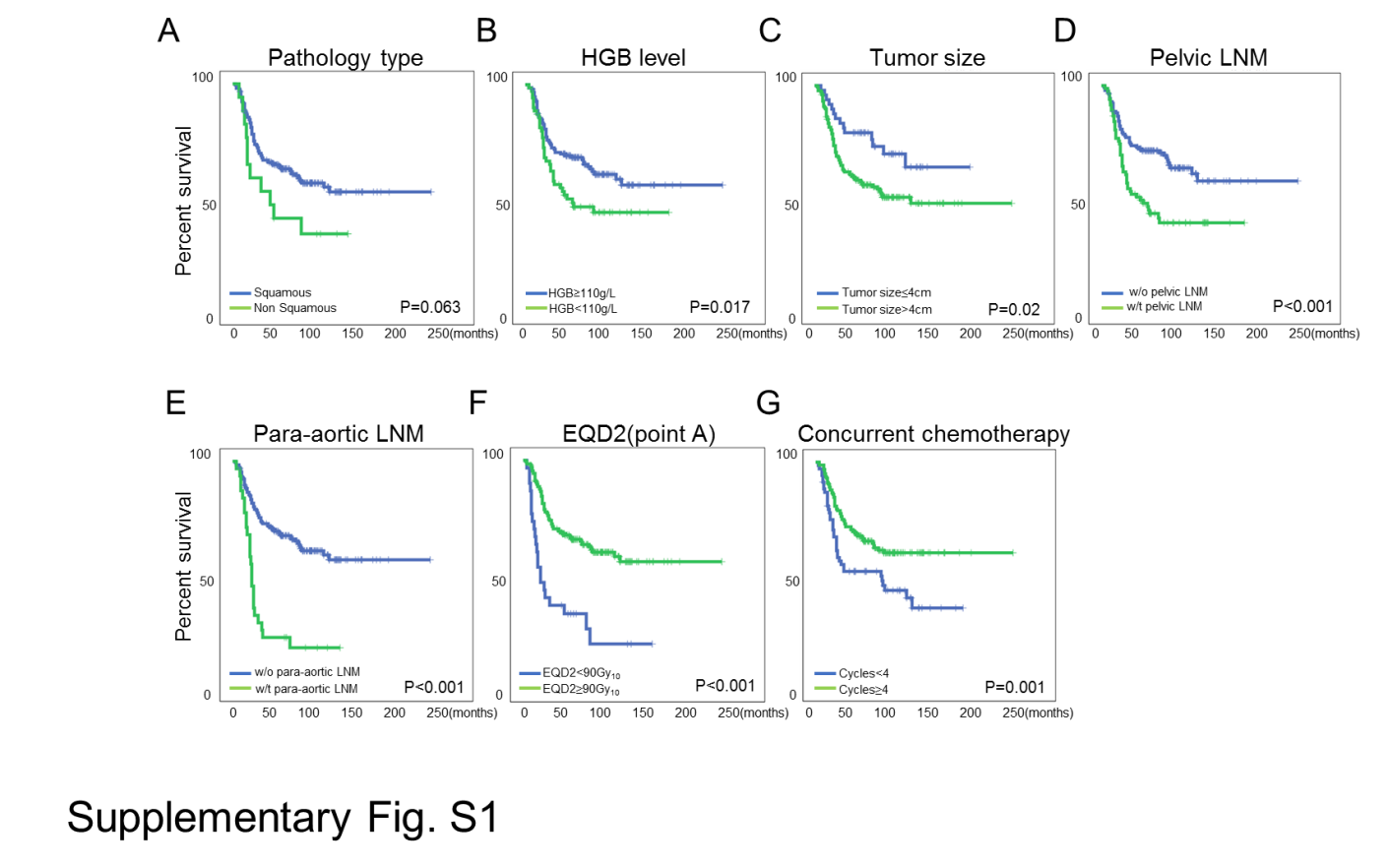


Figure S1
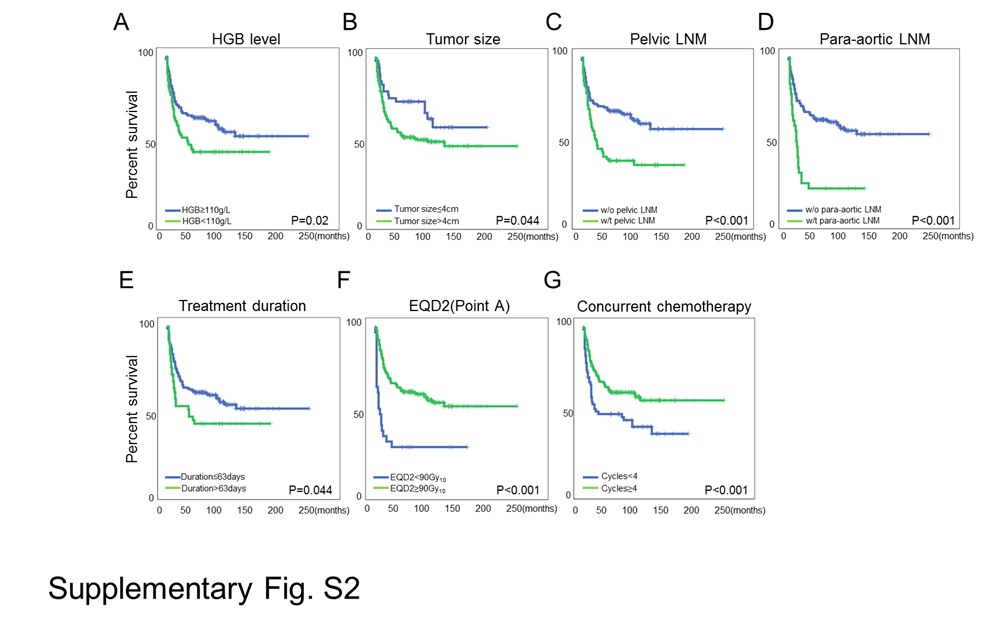


Figure S2


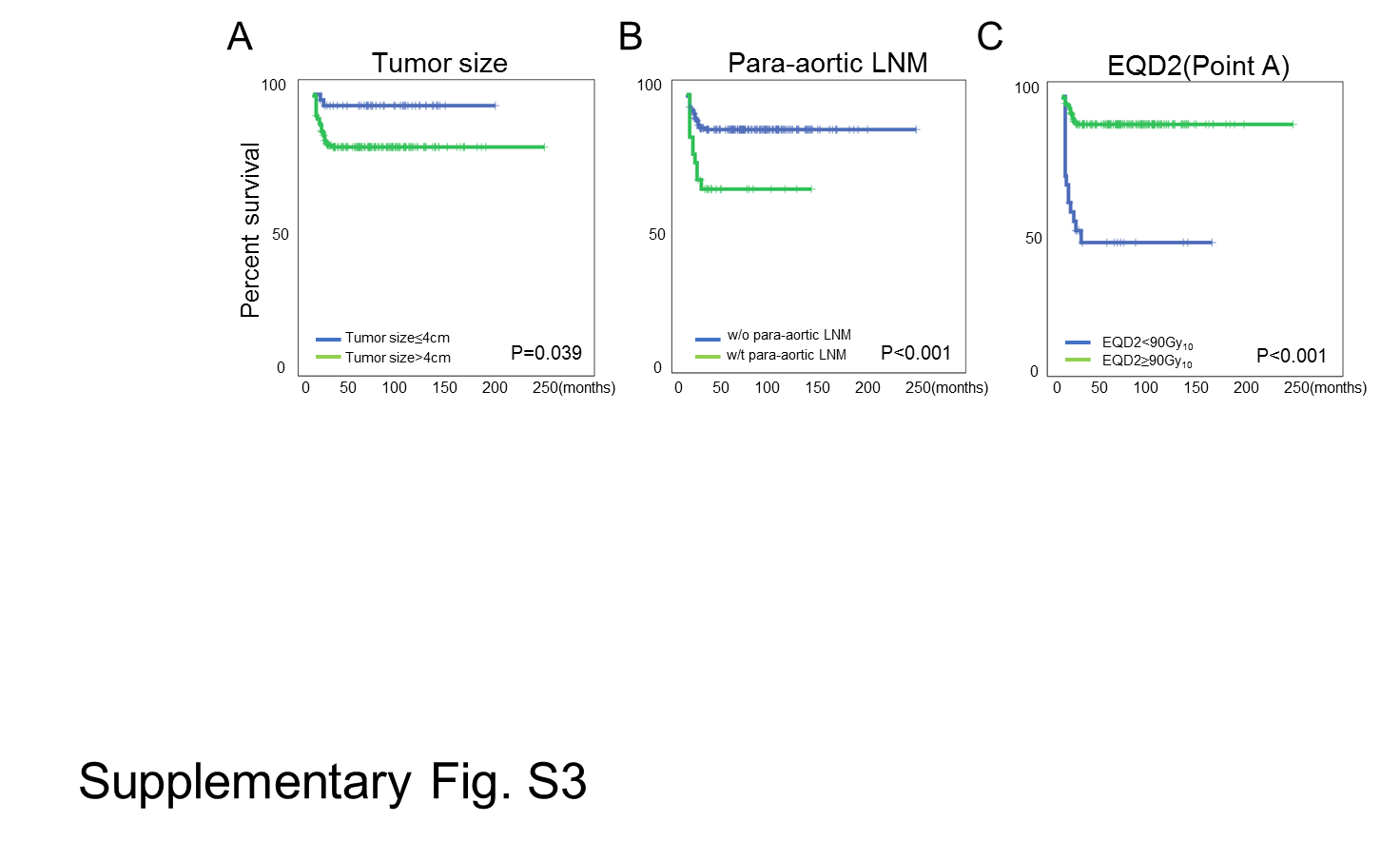


Figure S3


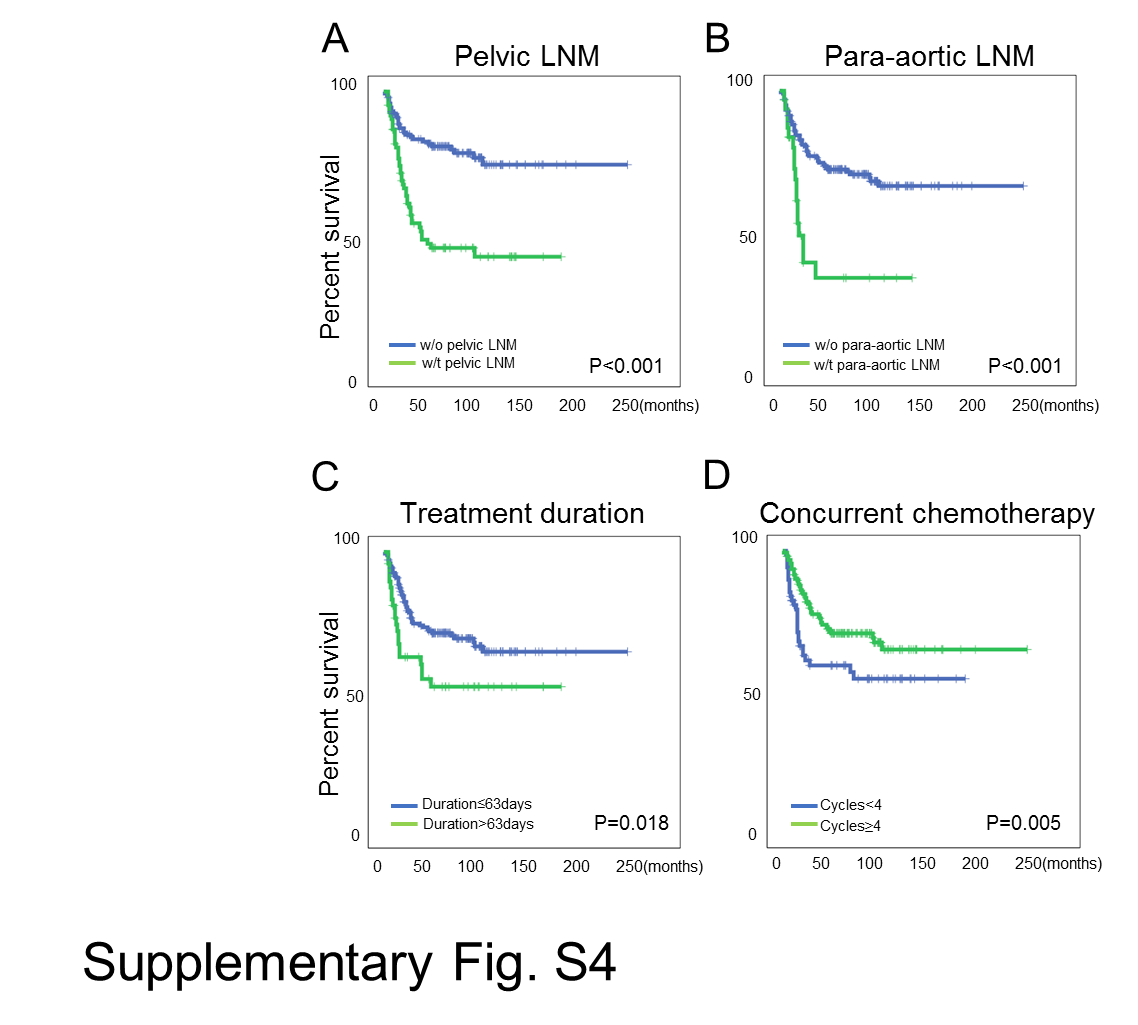


Figure S4
